# The effect of antibacterial and non-antibacterial compounds alone or associated with antifugals upon fungi

**DOI:** 10.3389/fmicb.2015.00669

**Published:** 2015-07-03

**Authors:** Maria M. Azevedo, Rita Teixeira-Santos, Ana P. Silva, Luisa Cruz, Elisabete Ricardo, Cidália Pina-Vaz, Acácio G. Rodrigues

**Affiliations:** ^1^Department of Microbiology, Faculty of Medicine, University of PortoPorto, Portugal; ^2^Center for Research in Health Technologies and Information Systems, Faculty of Medicine, University of PortoPorto, Portugal; ^3^School D. Maria IIVila Nova de Famalicão, Portugal; ^4^Department of Microbiology, Hospital São JoãoPorto, Portugal; ^5^Burn Unit, Department of Plastic and Reconstructive Surgery, Hospital São JoãoPorto, Portugal

**Keywords:** fungal infections, antibiotic therapy, antifungal therapy, combined therapy, synergistic effects

## Abstract

During the last 30 years the incidence of fungal infections has increased dramatically. While the antifungal therapeutic options available are somewhat reduced, most pathogenic microorganisms have an incredible capacity to mutate and acquire resistance. In addition, multiple drugs are often required concomitantly to manage clinically complex disorders. The combination of antibiotics or other compounds with antifungal drugs, simultaneously or sequentially, is commonly adopted in clinical practice, although without a full knowledge of the consequences. Thus, the role of combined therapy and the effect of antibiotics upon fungal growth promotion need to be critically evaluated and understood in order to avoid undesirable drug interactions. With this review we intend discuss the studies that report about antibiotics inhibiting fungal growth, as well as studies describing the synergistic effect of the combined therapy, i.e., associations between antibiotics or other compounds with antifungal drugs. Alternative therapeutic protocols for fungal disease could be designed, taking advantage of such drug combinations. Critical revision of previously published data is crucial in order to define future research strategies.

## Introduction

An increasing number of pathogenic microorganisms develop resistance to antimicrobial agents commonly used in medical therapy. Antibiotics are the most frequently prescribed antimicrobial drugs. These compounds reduce human and animal morbidity and mortality, providing the current basis for infectious disease control. However, soon after the introduction of penicillin (1943), reports about bacterial resistance started. The three most relevant causes of resistance are, (i) lack of the drug target, (ii) activity of chromosomally encoded antibiotic inactivating enzymes, and (iii) diminished uptake of the antimicrobial due to impermeability or eﬄux pumps.

On the other hand, the incidence of fungal infections during the last 30 years has increased dramatically; however, the number of antifungal compounds remains somewhat restricted. Several reasons were appointed to justify this increase such as the growing number of individuals with impairment of immune status, the increase of gastro-intestinal surgery and other invasive procedures, the growing number of patients admitted to intensive care units (ICUs), and the widespread use of wide spectrum antibacterial therapy.

It is essential to stress that systemic *Candida* infections are an important cause of morbidity and mortality among patients submitted to prolonged antibiotic therapy. The rate of bloodstream infection by *Candida* sp. in hospital patients increased about 500% along the 1980s, and 6–11% of all the positive nosocomial bloodstream infections could be attributed to *Candida* sp. (Pfaller and Diekema, 2007). *C. albicans*, like several other *Candida* species can inhabit humans as either commensal or opportunistic pathogens, being able to cause from superficial to life threatening infections. Treatment of fungal infections during the last 50 years has mostly been based upon fungicidal drugs such as amphotericin B, that binds to the major fungal sterol, ergosterol. Interestingly, the majority of clinical cases of antifungal resistance reported involved the azole class. A more recent group of drugs, the echinocandins, were described as fungicidal to yeast by inhibiting the cell wall synthesis. However, natural and acquired resistance was soon described (Walker et al., 2010).

It is important to highlight the fact that patients at risk for invasive fungal infections are also at risk for the development of serious bacterial infections; therefore, both antibacterial and other compounds may be used for prophylactic and therapeutic purposes in parallel or in sequence with antifungal drugs (Stergiopoulou et al., 2009).

In order to understand the effect of antibacterial and other compounds on fungal growth and on antifungal response, we conducted a review on this topic. This review was organized accordingly the following critical topics:

(1) Effect of antibacterial compounds:
  (a) Upon fungal growth
  (b) In association with antifungal drugs
(2) Effect of non-antibacterial drugs on antifungal therapy.

### 1(a) Effect of Antibacterial Compounds upon Fungal Growth

Data from literature revealed that the risk of infection by *Candida* sp. during and after antibiotic therapy was early recognized after the use of antibiotics. An ancient work conducted by Maclean (1962) suggested that the incidence of clinical candidosis may increase in patients treated with tetracycline, and further research showed that oral antibiotics administered in children, such as tetracycline and phenoxymethyl penicillin, induced the growth of *Candida* sp. It is well established that mucocutaneous candidosis is associated with antibacterial therapy. A pilot study demonstrated that the use of short courses of oral antibiotics (4–6 weeks) seems to increase the prevalence of asymptomatic vaginal *Candida* colonization as well as the incidence of symptomatic vulvovaginal candidosis (Xu et al., 2008). These results are consistent with previous studies which demonstrated that antibiotic use seems to be a short-term risk factor for symptomatic vulvovaginal candidosis and that highest risk occurs during the first month after antibiotic use.

*Candida* species await any opportunity afforded by antibacterial therapy; infections by yeast organisms often follow a course of antibiotic therapy. It is noteworthy that *Candida* sp. can show overgrowth following exposure to the majority of the antibiotics mostly by indirect effects that is decrease microbial competition. However, quinolones and aminoglycosides do not induce candidosis as promptly or as often as cephalosporins (Samonis et al., 1994). Cephalosporins are not active against all bacterial isolates. In consequence, microorganisms that are not inhibited by cephalosporin show overgrowth with varying potential to cause infection. Cephalosporins are prescribed for an extensive variety of infections, exhibiting broad spectrum activity, what encourages rapid overgrowth of some microorganisms that are neither eliminated nor inhibited. Overgrowth is not exclusively a bacterial domain. Among the panoply of such microorganisms, some are recognizable as pathogens; however, others that at the beginning may behave as commensal or seem to be of low risk status, have subsequently been shown to cause disease.

Mulligan et al. (1982) revealed that cefoperazone induced severe changes in fecal flora, such as suppression of *Enterobacteriaceae*, and increase in counts of *Candida* sp. A study involving a group of persons treated with parental ceftriaxone also showed an overgrowth of yeasts in stools; in patients treated with cefotaxime, *Candida* sp. was also recovered from throat swabs (Devrieshospers et al., 1991).

Pecquet et al. (1987) described a situation of five healthy adults receiving oral ofloxacin during 5 days (Pecquet et al., 1987); ofloxacin induced selective elimination of aerobic Gram-negative bacteria. During treatment, colonization of all the volunteers by *Candida* sp. was detected. The effect of amoxicillin on the ecology of skin microbial flora was also investigated; it revealed a decrease in the number of bacterial isolates and an increase in *C. albicans*. Samonis et al. (1994), revealed that amoxillin-clavulanate induced a higher and more persistent gastrointestinal colonization by yeasts comparatively to other tested antibiotics (Samonis et al., 1994). The effect of amoxicillin therapy on the ecology of skin microbial flora in infants was also investigated; it revealed a decrease in the number of bacterial isolates and increase in *C. albicans* recovery (Brook, 2000).

Among ICU patients infections by *Candida* sp. are of very high prevalence. A study performed by Charles et al. (2005), demonstrated that broad-spectrum antibiotic therapy was found to promote fungal growth in patients with prior yeast colonization (Charles et al., 2005). The authors recommended the reduction of antibiotic therapy for prevention of fungal infections in this particular case, since most cases of invasive candidosis in ICU setting are supposed to be subsequent to colonization in high-risk patients. A study involving ICU patients in Ankara Training and Research Hospital, Turkey, showed that *Candida* colonization emerged more often in patients with bacterial sepsis and in those exposed to broad spectrum antibiotics (Ergin et al., 2013). A study concerning fungaemia in a portuguese university hospital, revealed that 93% of the patients had received antibacterial drugs and in 68% of the cases, a combination of two or more antibacterial drugs had been administered simultaneously (Costa-de-Oliveira et al., 2008).

Another study involving antibiotic treated and untreated Syrian hamsters inoculated intragastrically with *C. albicans* showed that antibiotic treatment decreased the total population levels of the indigenous bacterial flora and predisposed the animals to gastrointestinal overgrowth and systemic dissemination of *C. albicans* in 86% of the cases (Kennedy and Volz, 1985a). These researchers proposed that the indigenous microflora inhibited *C. albicans* colonization and dissemination from the intestinal tract by two possible mechanisms: (i) decrease of the size of *Candida* population in the gut and, (ii) inhibition of the mucosal binding of *Candida* organisms by thick layers of bacteria in the mucus gel covering the epithelium. This study appears to suggest that anaerobic organisms suppress *C. albicans*. Hamsters treated with penicillin showed a decrease of the population of strictly anaerobic bacteria in the cecum, allowing an increase in facultative bacteria and promoting *C. albicans* adhesion, colonization, and dissemination from the gastrointestinal tract.

In a previous study involving mice treated with several antibiotics administered orally, namely penicillin, clindamycin, and vancomycin, a decrease in the total anaerobic bacterial populations in the animals ceca was found; simultaneously *C. albicans* could proliferate in the gut and subsequently disseminate to visceral organs (Kennedy and Volz, 1985b). In addition, it was hypothesized that presence of bacterial amines produced by anaerobes in vagina flora, as in healthy gut, may explain why candidosis is rarely observed in concomintance with bacterial vaginosis since such amines cause the inhibition of germ tube formation by *Candida* species (Rodrigues et al., 1999).

The perceived inhibitory effect of anaerobes upon yeasts, although by unknown mechanisms, is one of the stressed topics by authors like Stoutenbeek et al. (1984), when proposing selective digestive decontamination (SDD) protocols as a means of prevention of infection in patients admitted in ICUs (Stoutenbeek et al., 1984).

In a study involving a mouse model receiving single carbapenems (meropenem, imipenem, and ertapenem) or a carbapenem in association with amikacin, a substantial increase in the murine intestinal concentration of *C. albicans* was found (Samonis et al., 2013). Similar findings were found in humans. The level of gastrointestinal colonization by *Candida* sp. is related to the spectrum, the dose, the route of administration, and pharmacodynamics and pharmacokinetic properties of the antimicrobial given.

All these studies are summarized in **Table 1**. Notably, few studies were carried out, often with limited sample size were performed.

**Table 1:** Antibiotic drugs regimens that promote fungal growth mainly due to an indirect effect.

Class of antibiotics	Fungal organisms	Model of study	Sample size	Reference
Tetracycline	*Candida* sp.	*in vivo*^1^	unknown	Maclean (1962)
Associated with phenoxymethyl penicillin	*Candida* sp.	*in vivo*^1^	unknown	Maclean (1962)
Carbapenems	*C. albicans*	*in vivo*^2^	50	Samonis et al. (2013)
Associated with amikacin	*C. albicans*	*in vivo*^2^	50	Samonis et al. (2013)
Cephalosporins	*Candida* sp.	*in vivo*^1^	4	Mulligan et al. (1982)
	Yeasts	*in vivo*^1^	11	Devrieshospers et al. (1991)
Fluoroquinolone	*Candida* sp.	*in vivo*^1^	5	Pecquet et al. (1987)
Penicillins	*C. albicans*	*in vivo*^1^	25	Brook (2000)
	*Candida* spp.	*in vivo*^3^	unknown	Kennedy and Volz (1985b)
Associated with clavulanate	Yeasts	*in vivo*^1^	4	Samonis et al. (1994)
Glycopeptides	*Candida* sp.	*in vivo*^3^	unknown	Kennedy and Volz (1985b)
Aminoglycosides	*Candida* sp.	*in vivo*^3^	unknown	Kennedy and Volz (1985b)
Broad-spectrum antibiotic therapy	*Candida* sp.	*in vivo*^1^	593	Charles et al. (2005)
	*Candida* sp.	*in vivo*^1^	100	Ergin et al. (2013)

*^1^Human; ^2^Mouse model; ^3^Syrian hamster model.*

The explanation for antibiotic enhancement of yeast growth is mainly indirect, being related with: (i) removal of organisms competing for nutrients; and (ii) removal of organisms that secrete antifungal substances. However, a possible direct effect of antibiotic drugs in yeasts should be investigated.

### 1(b) Effect of Antibacterial Compounds in Association with Antifungal Drugs

It is common in clinical practice the use of antibiotics and antifungals simultaneously or sequentially. Thus, it is important to assess the effect of combination therapy on fungal cells.

Since the 1970s and 1980s sinergistic effects between tetracycline and amphotericin B were described. Tetracycline can change the susceptibility pattern of *C. albicans* and other pathogenic fungi to amphotericin B (Oliver et al., 2008). Tetracycline has a direct effect upon the mitochondrial function; its inhibition eliminates the diauxic shift and in consequence impairs sterol metabolism, which results in lower ergosterol levels. Lower sterol levels in cells grown in presence of tetracycline, promotes amphotericin B susceptibility due to a higher amphotericin B to ergosterol ratio at the cell surface (**Table 2**).

**Table 2:** Sinergistic effect between antibiotic and antifungal drugs.

Antifungals	Antibiotics	Fungal organisms	Reference	Mechanism of synergism
AMB	Tetracycline	*Candida albicans, Aspergillus fumigatus* and *Cryptococcus neoformans*	Oliver et al. (2008)	Inhibition of the mitochondrial function impairs sterol metabolism, resulting in lowe ergosterol levels.
	Azithromycin	*Fusarium* sp.	Clancy and Nguyen (1998)	Fungal cell membrane damaged by amphotericin B, may allow entrance of antibiotic, inhibiting protein synthesis.
	Rifampicin	*C. albicans*	Ansehn et al. (1976) Del Pozo et al. (2011)	
		*C. neoformans* and *Prototheca* sp.	Srimuang et al. (2000)	
		*C. parapsilosis, C. krusei* and *C. glabrata*	El-Azizi (2007)	
	Doxycycline	*C. parapsilosis, C. krusei*, and *C. glabrata*	El-Azizi (2007)	
	Clarithromycin	*Candida* sp.	Del Pozo et al. (2011)	
	Quinolone	*Candida* sp. and *C. neoformans*	Nakajima et al. (1995)	
FLU	Doxycycline	*C. albicans*	Gao et al. (2013)	Inhibition of protein synthesis may interfere with sterol pathway, resulting in lower ergosterol levels.
		*Candida* sp.	Miceli et al. (2009)	
	Tetracycline	fluconazole-resistant *C. albicans*	Shi et al. (2010)	
	Quinolone	fluconazole-resistant *C. albicans*	Sugar et al. (1997)	
	Tigecycline	*C. albicans* biofilms	Ku et al. (2010)	

*AMB, Amphotericin B; FLU, Fluconazole.*

Clancy and Nguyen (1998), investigated the combination of amphotericin B and azithromycin, against 26 clinical isolates of *Fusarium*, and observed a synergistic effect (Clancy and Nguyen, 1998). The combination of amphotericin B and azithromycin improved the antifungal effect reducing amphotericin B minimal inhibitory concentrations (MICs) from 1 to 0.37 mg/L, while single azithromycin exhibited no antifungal activity. Considering the resistance *of Fusarium* to conventional antifungal therapy, the combination of these two compounds could represent an important alternative strategy in fusariosis therapy (Clancy and Nguyen, 1998). Azithromycin acts by inhibiting protein synthesis. An advantage related to the use of azithromycin might arise from the excellent tissue levels that can be achieved. While the mechanism of synergism between these two compounds remains unclear, it has been postulated that amphotericin B, by damaging the fungal cell membrane, may facilitate the entrance of azithromycin into the cells; once inside the cells azithromycin might act by inhibiting fungal protein synthesis.

Data already published in 1976, demonstrated that amphotericin B combined with rifampicin impaired growth of *C. albicans*, and a pronounced fungistatic effect was obtained with therapeutically attainable concentrations of both these drugs (Ansehn et al., 1976). Moreover, it was demonstrated that rifampicin alone impaired the growth of this species.

Srimuang et al. (2000), evaluated the effect of amphotericin B alone and combined with rifampicin against 71 isolates of *Cryptococcus neoformans*, demonstrating that this combination resulted in synergistic effect.

It has been demonstrated that non-albicans *Candida* species (NAC), considered both colonizers, and pathogens, can cause serious nosocomial bloodstream infections. Moreover, 35–65% of all the candidemias were such species (Krcmery and Barnes, 2002), and there is a tendency to its increase. El-Azizi (2007), demonstrated that treatment with amphotericin B associated with rifampicin or doxycycline enhanced the killing activity of the antifungal agent in biofilms of *Candida parapsilosis, C. krusei*, and *C. glabrata* (El-Azizi, 2007). Antifungal activity of amphotericin B increased about 30–35% in presence of doxycycline; a synergistic effect was observed with concentrations of 512 mg/L of doxycycline. It is noteworthy that the tested antibiotics alone did not exhibit antifungal activity. The synergistic effect of the antibiotics could be related to the fact that amphotericin B binds to the sterols in the fungal cell membrane, thus increasing the antibacterial permeability; rifampicin interferes with RNA synthesis, while doxycycline interferes with protein synthesis.

A recent study has also described synergistic effects between amphotericin B and clarithromycin, and between amphotericin B and rifampicin against *Candida* biofilms. Attending to such results, the association between the antibacterial agents tested could represent a very interesting therapeutical approach for the treatment of *Candida* biofilm-related infections (Del Pozo et al., 2011).

Gao et al. (2013) explored the antifungal activity of fluconazole in combination with doxycycline against *C. albicans*. The results revealed a strong synergism against planktonic cells of fluconazole-resistant isolates. The same author’s also demonstrated a weaker antifungal effect of fluconazole combined with doxycycline against *C. albicans* biofilms compared with planktonic cells, being this effect time dependent. Miceli et al. (2009) also showed that doxycycline combined with fluconazole increased the killing activity of the antifungal upon biofilms by *Candida* sp. In this report, single doxycycline (2048 and 1024 mg/L) resulted in to 85% reduction of the metabolic activity of *C. albicans* biofilm; single fluconazole exhibited a lower activity (22.9 % reduction); interestingly, the combination of both compounds resulted in an additive effect. Such results suggest that high-dose doxycycline in combination with standard antifungal agents might play an important role in the treatment of *Candida* biomaterial-related, like is the case of medical indwelling device infections.

A recent study showed that fluconazole acts synergistically with minocycline against fluconazole-resistant *C. albicans*, resulting in a significant decrease of the MIC value when both compounds are combined comparatively to single fluconazole (Shi et al., 2010). This combination could represent a valid option to overcome fluconazole resistance. The minimum inhibitory concentrations for 80% of inhibition (MIC_80_) of fluconazole and minocycline were 512 and 256 mg/L, respectively. The results revealed that a concentration of 4 mg/L of minocycline (which corresponds to a concentration easily achieved in human serum and other body fluids with conventional therapeutic dosages), in combination with fluconazole resulted in a fluconazole MIC decrease to 8 mg/L. Data from the literature clearly demonstrate that it is very difficult for fluconazole to penetrate *C. albicans* biofilms when used alone. However, with the association of minocycline the amount of fluconazole penetrating *C. albicans* biofim increases significantly (Shi et al., 2010). The same study also showed that the association of minocycline with fluconazole could significantly reduce cell growth and cell activity. At a concentration of minocycline of 16-32 mg/L, the MIC of fluconazole decreased to 1–2 mg/L. In another study, tigecycline at concentrations corresponding to 2048 mg/L inhibited the growth of planktonic cells of *C. albicans* in 50% (Ku et al., 2010). Moreover, another study reported about an investigational quinolone that increased the activity of amphotericin B and fluconazole both *in vitro* an *in vivo* against a variety of fungal planktonic cells (Nakajima et al., 1995). Sugar et al. (1997) demonstrated that fluconazole was effective together with trovafloxacin in the treatment of mice infected with fluconazole-resistant *C. albicans* (Sugar et al., 1997). Such result suggests that combination therapy may have clinical potential.

Since the ability of microorganisms to form biofilms is often involved in severe infections, with the consequent emergence of resistance to the host immune system response and to antimicrobial therapy, it is important to emphasize that antibacterial compounds alone or combined with antifungals could also play a relevant role against fungal biofilms. A study developed by Ku et al. (2010) showed that 2048 mg/L of TIG reduced the metabolic activity of mature biofilms in 84.2%; the association of 512 mg/L of TIG with fluconazole, at all the tested concentrations, resulted in an extra reduction of the metabolic activity of mature biofilms (Ku et al., 2010).

A possible explanation for the synergistic effects between fluconazole and antibiotics that act by inhibition of RNA or protein synthesis could be related with the inhibition of protein synthesis, which may interfere with sterol pathway ultimately, decreasing the ergosterol levels. Details about the most relevant studies addressing synergistic effects between antibiotic and antifungal drugs are detailed in **Table 2**.

## 2. Effect of Non-Antibacterial Drugs on Antifungal Therapy

Only synergistic effects between antifungals and other chemical compounds, other than, classic antibiotics were reviewed; details are described in **Table 3**.

**Table 3:** Sinergistic effect between other non-antibacterial compounds and antifungal drugs.

Antifungals	Other compounds	Fungal organisms	Reference	Mechanism of synergism
Azoles	Chloroquine	*C. albicans*	Shinde et al. (2013)	Iron deprivation
		*S. cerevisiae*	Emerson et al. (2002)	
	Ibuprofen	*C. albicans*	Pina-Vaz et al. (2000)	Eﬄux pump blocker; increase of azole content
	Lactoferrin	*Candida* sp.	Bellamy et al. (1993)	Direct interaction with the cell surface
			Kuipers et al. (1999)	
	*Sida Cordifolia* compounds	*Candida* sp.	Ouédraogo et al. (2012)	Immunostimulatory effect
	Sertraline	*Candida* and *Cryptococcus* sp.	Spitzer et al. (2011)	Alteration of membrane permeability
	Statins	*Dermatophyte* sp.	Nyilasi et al. (2014)	Not described
AMB	Lactoferrin	*Candida* sp.	Bellamy et al. (1993)	Direct interaction with the cell surface
	Statins	*Dermatophyte* sp.	Nyilasi et al. (2014)	Not described
5-FC	Lactoferrin	*Candida* sp.	Bellamy et al. (1993)	Direct interaction with the cell surface
Nystatin	*Sida Cordifolia* compounds	*Candida* sp.	Ouédraogo et al. (2012)	Immunostimulatory effect

*AMB, Amphotericin B; 5-FC, 5-fluorocytosine.*

Chloroquine exhibits antifungal activity against fungal pathogens. Shinde et al. (2013) revealed that chloroquine potentiates the anti-biofilm activity of fluconazole and voriconazole upon *C. albicans*. A study involving *Saccharomyces cerevisiae* also demonstrated that chloroquine inhibits the yeast growth by iron deprivation (Emerson et al., 2002). While similar effects could occur with *C. albicans* in presence of fluconazole and voriconazole, the precise mechanism remains yet to be elucidated (Shinde et al., 2013).

Alem and Douglas (2004) showed that aspirin also exhibited antibiofilm activity *in vitro*; in combination with antifungal agents it could be used against biofilm associated *Candida* infections (Alem and Douglas, 2004). A study performed at our laboratory revealed that a combination of fluconazole with sodium salicylate or ibuprofen induced a synergistic activity against *C. albicans*, due to blockage of eﬄux pumps (Pina-Vaz et al., 2000). Bellamy et al. (1993) demonstrated that lactoferricin B exhibits fungicidal effect upon *Candida* species through direct interaction with the cell surface. Kuipers et al. (1999) showed a synergistic activity between lactoferrin and amphotericin B, 5-fluorocytosine and fluconazole against *Candida* species. However, the most successful combination was lactoferrin and fluconazole.

A recent report describes synergistic interactions between statins and antifungals (Nyilasi et al., 2014). Authors suggest that statins exhibit antifungal effect against dermatophyte fungi, which could constitute promising compounds for combination therapy.

The search for plants with antifungal activity has gained increasing importance; higher plants produce diverse secondary metabolites with different biological activities. These compounds may be exhibit antimicrobial effects. Ouédraogo et al. (2012) noticed that alkaloid compounds from *Sida Cordifolia L* (*Malvaceae*) in combination with nystatin and clotrimazole exhibited inhibitory effects against *Candida* strains. A hypothesis for this effect can be related with alteration of membrane permeability.

The combination of fluconazole with sertraline, an antidepressant, was effective against *Candida* and *Cryptococcus* strains, including drug-resistant clinical isolates of *Candida*, in an *in vivo* model (Spitzer et al., 2011). Such result may be related with concomitant immunostimulatory effect.

## Conclusion

While the interaction between different antimicrobials and with other compounds still remains unclarified in certain domains, there is certainly place for additional and more comprehensive studies addressing additive and synergistic effects between antibacterials and antifungals, in particular, including preliminary clinical trials. The rationale for such studies arises from the clinical relevance that fungal pathogens have assumed during the recent past years, the economic and social burden that fungal infections represent for public health systems and the inherent therapeutic challenges, namely in terms of safety and high efficacy antifungal regimens. Additional studies, in particular addressing the topic of combination therapy, are highly needed to provide sound scientific support for the clinical association of classic antifungals and non-conventional antifungals drugs. Research in this field may ultimately provide valuable solutions for such demanding societal challenges.

Regarding future research we now come forward with several suggestions that would help to elucidate questions related to the controversial topics discussed in this review. First, it is crucial to understand whether the stimulus for fungal growth is related to the inhibition of competition between fungi and bacteria or if the antibacterial compounds could act directly upon fungal cells. Simultaneously, it is crucial to pursue experiments in order to study the effect between major antifungal classes – azoles, echinocandins and polyenes – and antibiotics that act impair RNA/protein synthesis and target the cell membrane, since data from literature suggested that these are the most promising combinations. This kind of studies should be involve to a significant wide range of species and isolates, since in most of the studies described above, samples sizes were invariably reduced. Ultimately, such results would support the design of novel therapeutic guidelines for antimicrobial use (alone and in association), aiming to prevent or treat invasive fungal infections. This issue is particularly important to ensure therapeutic success and to limit the emergence of antifungal resistance.

## Author Contributions

MA, CV, and AR: design of the study; MA and RS drafted the work; MA, RS, and AS wrote and revised the manuscript; LC, ER, CV, and AR revised the final version to be published.

## Conflict of Interest Statement

The authors declare that the research was conducted in the absence of any commercial or financial relationships that could be construed as a potential conflict of interest.
